# Comparison of Four Severity Assessment Scoring Systems in Critically Ill Patients for Predicting Patient Outcomes: A Prospective Observational Study From a Single Tertiary Center in Central India

**DOI:** 10.7759/cureus.66268

**Published:** 2024-08-06

**Authors:** Subhendu Mishra, Alok K Swain, Santosh Tharwani, Devendra Kumar, Shilpa Meshram, Ankit Shukla

**Affiliations:** 1 Anaesthesiology and Critical Care Medicine, Balco Medical Centre, Raipur, IND; 2 Oncoanesthesiology, Balco Medical Centre, Raipur, IND; 3 Critical Care Medicine, Amar Jain Hospital, Jaipur, IND

**Keywords:** discrimination, calibration, predictive accuracy, sofa, rems, saps ii, apache ii, intensive care unit

## Abstract

Background and aim

A variety of scoring systems are employed in intensive care units (ICUs) with the objective of predicting patient morbidity and mortality. The present study aimed to compare four different severity assessment scoring systems, namely, Acute Physiology and Chronic Health Evaluation II (APACHE II), Rapid Emergency Medicine Score (REMS), Sequential Organ Failure Assessment (SOFA), and Simplified Acute Physiologic Score II (SAPS II) to predict prognosis of all patients admitted to a mixed medical ICU of a tertiary care teaching hospital in central India.

Methods

The prospective observational study included 1136 patients aged 18 years or more, admitted to the mixed medical ICU. All patients underwent severity assessment using the four scoring systems, namely APACHE II, SOFA, REMS, and SAPS II, after admission. Predicted mortality was calculated from each of the scores and actual patient outcomes were noted. Receiver operating curve analysis was undertaken to identify the cut-off value of individual scoring systems for predicting mortality with optimum sensitivity and specificity. Calibration and discrimination were employed to ascertain the validity of each scoring model. Bivariate and multivariable logistic regression analyses among the study participants were conducted to identify the best scoring system, after adjusting for potential confounders.

Results

Final analysis was done on 957 study participants (mean (±SD) age-58.4 (±12.9) years; males-62.2%). The mortality rate was 14.7%. APACHE II, SOFA, SAPS II, and REMS scores were significantly higher among the non-survivors as compared to the survivors (p<0.05). SAPS II was found to have the highest AUC of 0.981 (p<0.001). SAPS II score >58 had 93.6% sensitivity, 94.1% specificity, 73.3% PPV, 98.8% NPV, and 94.0% diagnostic accuracy in predicting mortality. This scoring system also had the best calibration. Binary logistic regression showed that all four scoring systems were significantly associated with ICU mortality. After adjusting for each other, only SAPS II remained significantly associated with ICU mortality.

Conclusion

Both SAPS II and APACHE II were observed to have good calibration and discriminatory power; however, SAPS II had the best prediction power suggesting that it may be a useful tool for clinicians and researchers in assessing the severity of illness and mortality risk in critically ill patients.

## Introduction

In a tertiary care hospital, patients with medical and surgical conditions of variable severity are admitted to the intensive care unit (ICU). Increasing severity of the disease exacerbates organ dysfunction, leading to adverse effects on patient morbidity and mortality [1,2]. Diverse assessment systems have been implemented to ascertain the severity of diseases and predict the prognosis of such patients [2-5]. A multitude of investigations have been conducted to assess the predictability of scores pertaining to the severity of illnesses; thus far, contradictory findings have been documented [6]. Of the various severity assessment scoring systems, the most frequently used are Acute Physiology and Chronic Health Evaluation II (APACHE II), Rapid Emergency Medicine Score (REMS), Sequential Organ Failure Assessment (SOFA), and Simplified Acute Physiologic Score II (SAPS II) [2,3].

Knaus et al. introduced Acute Physiology and Chronic Health Evaluation II (APACHE II) in 1985, which was among the initial physiologic scoring systems. This model is computed using twelve physiological criteria, the patient's age, and their prior medical condition [5,7]. The Simplified Acute Physiologic Score II (SAPS II), proposed by Le Gall et al., comprises a total of 17 variables [8]. These variables include twelve physiologic parameters, age, type of admission, and three variables pertaining to underlying diseases [9]. The model's predictive capability has been validated across various clinical scenarios [10-12].

Among the emergency scoring systems, the Rapid Emergency Medicine Score (REMS) was modified by Olsson et al. from Rapid Acute Physiological Score (RAPS) in 2003 [13]. It is based on six readily obtained parameters in an emergency setting, including body temperature, oxygenation, and age, and is reported to be a strong predictor of in-hospital mortality [13-15]. The Sequential Organ Failure Assessment (SOFA) score, developed in 1994, is an objective and straightforward score that permits computation of both the number and severity of organ dysfunction in six systems (respiratory, coagulation system, liver, cardiovascular, renal, and neurologic); each system is assigned a score between 0 and 4, with a higher score indicating organ dysfunction that is deteriorating; the score can be used to quantify organ dysfunction individually or collectively [16,17].

Initially designed to assist ICU patients with sepsis, the aforementioned severity assessment scoring systems have since been applied to other critically ill patient populations, including those with community-acquired pneumonia in the elderly [18], sepsis patients admitted to the ICU, acute poisoning [19], and COVID-19 patients in the ICU [20]. Given that each system employs a unique combination of parameters to stratify the patient, comparing different scoring systems is crucial in order to ascertain which combination of parameters provides the most accurate prediction of the patient's condition. A multitude of investigations have been conducted to assess the reliability of these scoring systems; thus far, contradictory findings have been documented [21]. With this background, the present study aimed to compare the four most commonly employed severity assessment scoring systems, namely Acute Physiology and Chronic Health Evaluation II (APACHE II), Rapid Emergency Medicine Score (REMS), Sequential Organ Failure Assessment (SOFA), and SAPS II to predict prognosis of all patients admitted to a mixed medical ICU (composed of medical and post-operative patients) of a tertiary care teaching hospital in central India.

## Materials and methods

Study design and participants

The present prospective observational study was conducted in a tertiary care teaching hospital (NH MMI Hospital, Raipur) in Central India from July 2021 to December 2022 after approval from the Institutional Ethics Committee (vide reference no. 1631/IEC-AIIMSRPR/2021). A total of 1136 patients aged 18 years or more, admitted to the mixed medical ICU, were included in the study after obtaining written informed consent from the patient or their legal attendant (if the patient is not in a position to give consent). Pregnant patients and those who were lost to follow-up were excluded from the study.

Method of data collection

Upon admission to the ICU, all patients underwent severity assessment using the four scoring systems taken up in the study: APACHE II, SOFA, REMS, and SAPS II. Baseline demographics, including age, sex, reason for admission to the ICU, primary system involved, and comorbid conditions were collected on enrolment. Other vital parameters and data from routine blood investigations (CBC, LFT, KFT, ABG) were used for calculating the various scores. Based on the scores at admission, predicted mortality was calculated using a score-specific calculator available online (MedCalc). The patients were followed up throughout their stay in the ICU and the length of stay was recorded and the final outcome, whether discharged or dead was also noted. All details were recorded in a predesigned, pretested proforma.

Statistical analysis

Data collected was collated and entered in Microsoft Excel 2016 (Microsoft Corporation, Redmond) and statistical analysis was done using IBM SPSS Statistics for Windows, Version 24 (Released 2016; IBM Corp., Armonk, New York, United States). For each scoring system, observed mortality and predicted mortality (in different predicted mortality tiers) were tabulated and correlated. Receiver operating curve (ROC) analysis was undertaken to identify the cut-off value of the individual scoring system for predicting mortality with optimum sensitivity and specificity.

Calibration and discrimination were employed to ascertain the validity of each scoring model. The assessment of calibration, which refers to the extent to which predicted and observed mortality across all risks correspond, was conducted using calibration curves and the Hosmer-Lemeshow goodness-to-fit C statistic. Indeed, a model that possessed a lower Hosmer-Lemeshow value and a higher p-value (>0.05) was deemed superior. ROC curves were used to evaluate discrimination, which was defined as the capacity of the model to distinguish between patients who died and those who survived. The predictive performance of each scoring system was evaluated using the Z test, which compared the area under the curve (AUC) of the ROC curves. A higher AUC indicates superior predictive ability. Bivariate and multivariable logistic regression analyses among the study participants were conducted to identify the best scoring system, after adjusting for potential confounders. Variables with a p-value of <0.05 in bivariate analysis were included in the multivariable analysis. In all statistical analyses, a p-value less than 0.05 was deemed to indicate statistical significance.

## Results

The study included 1136 patients admitted to the mixed medical ICU of the hospital, out of which 179 patients were lost to follow-up (due to discharge against medical advice) and final analysis was done on 957 study participants. Out of them, the majority of the participants were aged between 60 and 75 years (44.9%), followed by those between 45 and 60 years (37%). Patients < 45 years constituted the least proportion of study participants. The mean (±SD) age of the study participants was 58.4 (±12.9) years; the median age was 60 years, and the minimum and maximum ages were 18 years and 91 years respectively. There was a male preponderance of patients admitted to the ICU (62.2%), with a male-to-female ratio of 1.64:1.

In our study participants, at ICU admission, the median (IQR) APACHE II score was 16 (10-25); the median SOFA score was 4 (2-8); the median SAPS II score was 32 (23-50) and the median REMS score was 8 (5-12). The proportion of patients who had 24-48 hours of stay in the ICU was 22.9% (n=219). Only 6.3% (n=60) of the patients were either discharged or died within 24 hours of ICU admission, while 22.8% (n=218) patients had an ICU stay of >5 days.

One hundred and forty-one participants died in the ICU, accounting for an ICU mortality rate of 14.7%. Relevant tests of significance revealed that while mean ± SD age, gender, and length of ICU was comparable between survivors and non-survivors; all four scores, namely, APACHE II, SOFA, SAPS II, and REMS scores, were significantly higher among the non-survivors as compared to the survivors (p<0.05) (Table 1).

**Table 1:** Comparison of age, gender, severity assessment scores, and length of stay between survivors and non-survivors (n=957) Values are presented as n (%) or mean ± SD ^#^Based on the Chi-square test for categorical data (gender) and Mann-Whitney U test for continuous data (age, APACHE II score, SOFA score, SAPS II score, REMS score, and length of ICU stay). p<0.05 is considered as statistically significant. APACHE II: Acute Physiology and Chronic Health Evaluation II; REMS: Rapid Emergency Medicine Score; SOFA: Sequential Organ Failure Assessment; SAPS II: Simplified Acute Physiologic Score II

Characteristics	Non-survivors (N=141)	Survivors (N=816)	t-value/ chi-square value	p-value^#^
Age (years)	59.5 ± 13.7	58.2 ± 12.8	1.084	0.279
Gender (male)	86 (61.0)	509 (62.4)	0.098	0.754
APACHE II score	37.7 ± 6.3	15.2 ± 8.1	31.520	<0.001
SOFA score	13.0 ± 2.8	4.1 ± 3.1	32.467	<0.001
SAPS II score	69.9 ± 8.3	32.0 ± 14.1	30.963	<0.001
REMS score	14.5 ± 2.1	7.3 ± 3.8	21.762	<0.001
Length of ICU stay (hours)	78.2 ± 77.3	90.7 ± 79.5	1.736	0.083

Table 2 tabulates the observed mortality versus mean of predicted mortality in different predicted mortality tiers for the four scoring systems. For the APACHE II scoring system, the mean predicted mortality was 32.8%. The mean predicted mortality, following the SAPS II scoring system, was 28.2%. In both APACHE II and SAPS II, predicted mortality best correlated with observed mortality in the highest tier (between 80-100% predicted mortality). For the SOFA scoring system, in the highest tier with predicted mortality>95%, the mean observed mortality was 85.7%; for predicted mortality of 50%, the mean observed mortality was 27.4%, while for predicted mortality <33%, the mean observed mortality was only 2.1%. For the REMS scoring system, the mean predicted mortality was 6.8%. Predicted mortality did not correlate well with the observed mortality in any of the tiers. In the predicted mortality tier of 20-40%, the mean observed mortality was 91.4%.

**Table 2:** Observed mortality versus mean of predicted mortality in different predicted mortality tiers for the four scoring systems (n=957) APACHE II: Acute Physiology and Chronic Health Evaluation II; REMS: Rapid Emergency Medicine Score; SOFA: Sequential Organ Failure Assessment; SAPS II: Simplified Acute Physiologic Score II

Predicted mortality tier	No. of patients	Observed deaths	Observed mortality (%)	Mean predicted mortality (%)
APACHE II score				
0-20	436	0	0	10.7
20-40	275	4	1.5	30.5
40-60	62	10	16.1	55.0
60-80	60	26	43.3	73.0
80-100	124	101	81.5	85.0
SOFA score				
<33	769	16	2.1	-
50	62	17	27.4	-
>95	126	108	85.7	-
SAPS II score				
0-20	563	0	0	7.3
20-40	133	3	2.3	29.7
40-60	67	5	7.5	50.5
60-80	97	42	43.3	71.5
80-100	97	91	93.8	88.4
REMS score				
0-20	897	87	9.7	4.6
20-40	58	53	91.4	38
40-60	0	0	0	0
60-80	2	1	50	75

**Figure 1:**
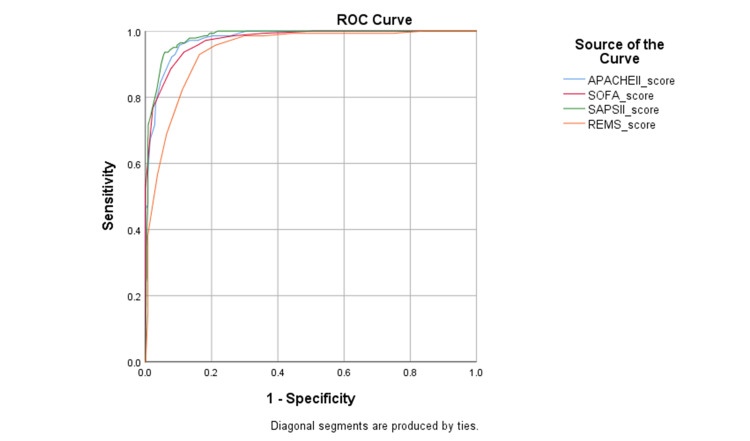
ROC curves for the different scoring systems on ICU admission in predicting mortality (n=957) APACHE II: Acute Physiology and Chronic Health Evaluation II; REMS: Rapid Emergency Medicine Score; SOFA: Sequential Organ Failure Assessment; SAPS II: Simplified Acute Physiologic Score II; ROC: Receiver Operating Characteristic

Figure 1 represents the ROC curves for the different scoring systems on ICU admission in predicting mortality.

Among all, SAPS II was found to have the highest AUC of 0.981 which was statistically significant. SAPS II score >58 had 93.6% sensitivity, 94.1% specificity, 73.3% PPV, 98.8% NPV, and 94.0% diagnostic accuracy in predicting mortality (Table 3 and Table 4).

**Table 3:** ROC analysis for the different scoring systems on ICU admission in predicting mortality (n=957) APACHE II: Acute Physiology and Chronic Health Evaluation II; REMS: Rapid Emergency Medicine Score; SOFA: Sequential Organ Failure Assessment; SAPS II: Simplified Acute Physiologic Score II; ROC: Receiver Operating Characteristic

Scoring system	Area under the curve (AUC)	Standard error (SE)	p-value	95% CI	
				Lower bound	Upper bound
APACHE II	0.976	0.005	<0.001	0.968	0.985
SOFA	0.973	0.006	<0.001	0.962	0.984
SAPS II	0.981	0.004	<0.001	0.973	0.989
REMS	0.940	0.009	<0.001	0.922	0.958

**Table 4:** Diagnostic test parameters of different scoring systems on ICU admission in predicting mortality (n=957) * This threshold is calculated based on the Youden index # Diagnostic test parameters could not be calculated for SOFA as predicted mortality was calculated as relative values instead of continuous numbers APACHE II: Acute Physiology and Chronic Health Evaluation II; REMS: Rapid Emergency Medicine Score; SAPS II: Simplified Acute Physiologic Score II; SOFA: Sequential Organ Failure Assessment; PPV: Positive Predictive Value; NPV: Negative Predictive Value

Scoring system^#^	Threshold*	Sensitivity	Specificity	PPV	NPV	Diagnostic accuracy
APACHE II	24	97.2	86.6	55.7	99.4	88.2
SAPS II	58	93.6	94.1	73.3	98.8	94.0
REMS	11	92.9	83.7	49.6	98.6	85.1

This scoring system was also found to have the best calibration as indicated by a lower goodness-of-fit value and highest p-value (Table 5).

**Table 5:** Goodness-of-fit Hosmer-Lemeshow test and p-value for the different scoring systems (n=957) APACHE II: Acute Physiology and Chronic Health Evaluation II; REMS: Rapid Emergency Medicine Score; SAPS II: Simplified Acute Physiologic Score II; SOFA: Sequential Organ Failure Assessment

Scoring system	Goodness-of-fit value	p-value	
			APACHE II	3.854	0.803	
SOFA	4.262	0.623	
SAPS II	3.275	0.845	
REMS	7.105	0.398	

Binary logistic regression showed that all four scoring systems, APACHE II, SOFA, SAPS II, and REMS, were significantly associated with ICU mortality. After adjusting for each other, only SAPS II remained significantly associated with ICU mortality (Table 6).

**Table 6:** Binary and multivariable logistic regression to determine factors associated with ICU mortality (n=957) OR: Odds ratio; AOR: adjusted odds ratio; APACHE II: Acute Physiology and Chronic Health Evaluation II; REMS: Rapid Emergency Medicine Score; SAPS II: Simplified Acute Physiologic Score II; SOFA: Sequential Organ Failure Assessment *p<0.05 is considered as statistically significant.

Parameters	OR (95% CI)	AOR (95% CI)	p-value
APACHE II score	1.38 (1.30-1.46) *	1.02 (0.91-1.13)	0.784
SOFA score	2.17 (1.90-2.49) *	1.17 (0.93-1.48)	0.170
SAPS II score	1.26 (1.20-1.31) *	1.21 (1.13-1.29) *	<0.001 *
REMS score	1.97 (1.77-2.20) *	1.12 (0.93-1.36)	0.231
Length of ICU stay	0.99 (0.98-1.01)	0.99 (0.98-1.02)	0.065

## Discussion

A variety of scoring systems are employed in ICUs with the objective of predicting patient morbidity and mortality. The present study compared four commonly used severity assessment scoring systems. Variations in ICU mortality rates are attributable to patient and institutional attributes. In our study, the rate of mortality in the ICU was 14.7%. This figure is similar to the 13.5% all-cause mortality rate in the ICU reported by Sekhar et al. [22] but is lower than the 26.5% mortality rate reported by Rahmatinejad et al. [23]. The observed mortality rate (14.7%) was lower than the mean predicted mortality by APACHE II (32.8%) and SAPS II (28.2%) in our study. This discrepancy is likely attributable to the quality of care provided during the ICU stay and the subsequent follow-up. At the highest stratum of predicted probabilities, both APACHE II and SAPS II indicated good agreement between actual and predicted ICU mortality probabilities. On the contrary, the SOFA exhibited a tendency to underestimate the mortality rate across all probabilities, whereas the REMS overestimated it in the highest tier of predicted probabilities and underestimated it in the lower tiers. A study by Rahmatinejad et al. [23] also reported the poor performance of SOFA in contrast to APACHE II and SAPS II which performed better in the calibration plots.

The present study evaluated and compared the discriminatory ability of the four scorings systems at the time of admission in predicting mortality. SAPS II was noted to have the best discriminatory ability with an AUC of 0.981 which was closely followed by APACHE II with an AUC of 0.976. A SAPS II score >58 predicted mortality with 93.6% sensitivity, 94.1% specificity, and 94% diagnostic accuracy; while an APACHE II score >24 predicted mortality with a sensitivity (97.2%) higher than SAPS II, but lower specificity (86.6%) and a lower diagnostic accuracy of 88.2%. In comparison to SAPS II and APACHE II, REMS value >11 had lower sensitivity (92.9%), specificity (83.7%), and diagnostic accuracy (85.1%) along with an imperfect calibration of the model as well. A study by Sekhar et al. [22] reported the AUC of SAPS II as 0.89 and a cut-off score of 53 predicting 63% of mortality cases; the AUC of APACHE II was 0.81 with a score >28 predicting 33.3% of the mortality; while AUC of REMS was 0.72 and a cut-off value of 10 predicted only 14.8% of the mortality. Another study by Aminiahidashti et al. [24] however established that both SAPS II and APACHE II had excellent calibration and discriminatory power, which were comparable with each other. Even studies conducted on COVID-19 patients reported SAPS II, APACHE II, and SOFA as effective tools in predicting mortality [25,26].

In the present study, a particular observation was practically experienced, wherein the patients admitted to the ICU with primary neurological etiology tended to have severity scores in the middle- or lower-tier but they tended to have a long stay in the ICU and either recovered or died after prolonged stay due to secondary complications. Moreover, it needs to be highlighted that most severity scoring systems take into account the presence of chronic health conditions or comorbidities as one of the various parameters (such as Chronic Health points in APACHE II and chronic disease in SAPS II). For the same reason, comorbidities among our study participants have not been considered in the regression model independently.

The findings of the present study established SAPS II to have the best discriminatory and predictive ability as proved by the score with the highest AUC and the only score to remain significant in multivariable logistic regression after adjusting for potential confounder. SAPS II is a well-established and widely used severity assessment score that includes 17 physiological variables, such as heart rate, blood pressure, and laboratory values, along with age and the presence of comorbidities. The score ranges from 0 to 163, with higher scores indicating greater severity of illness and higher mortality risk. In contrast, more recent scoring systems, namely the SAPS III and APACHE III used more than 200 variables in a complex algorithm to calculate the scores. We discourage the use of such scoring systems at the time of ICU admission, as it is not always practical in most hospital settings to have all parameters readily available within 24 hours of ICU admission. In terms of feasibility and simplicity of calculations, SAPS II outperforms the aforementioned advanced scoring systems.

Additionally, prior studies have shown that SAPS II is a reliable and accurate predictor of mortality in ICU patients [27]. The superior predictive performance of SAPS II in this study could have several explanations. One possible reason is that SAPS II includes a more comprehensive set of physiological variables and comorbidities than some of the other scores. This may provide a more nuanced and accurate assessment of a patient's overall health status and mortality risk. Another possible reason for the better predictive power of SAPS II is that it was originally developed and validated using a large and diverse cohort of ICU patients. This may have helped to ensure that the score is generalizable and applicable to a wide range of patient populations and clinical settings.

It is important to note, however, that no severity assessment score is perfect, and each has its limitations and potential sources of bias. In addition, clinical judgment and individual patient factors also play an important role in decision-making in the ICU. Therefore, while SAPS II may have shown the best prediction power in our study, it should be used in conjunction with other clinical information and not relied upon in isolation. In fact, it was observed that SAPS II better predicted mortality in the subset of patients admitted to the ICU with hepatobiliary aetiologies; which may be attributed to the presence of bilirubin as a parameter for evaluating the SAPS II score. Although the SOFA score also included bilirubin in its calculation, variations in weightage and exclusion of age as a parameter in SOFA and a combination of other associated parameters in both the scoring systems may have favored a reliable diagnosis for this subgroup of patients by the SAPS II scoring system.

However, just like any other scoring system, SAPS II also has some limitations. Firstly, although SAPS II has shown good overall predictive power, it may not be equally accurate in all patient populations. Further studies are warranted to validate the prognostic accuracy in certain subgroups of ICU patients, such as those with liver disease or traumatic brain injury. Secondly, SAPS II relies heavily on physiological variables, such as vital signs and laboratory values, to predict mortality risk. While these variables are important indicators of a patient's health status, they may not fully capture the complexity of some conditions or the influence of other factors, such as psychosocial factors or pre-existing comorbidities. Thirdly, as with any severity assessment score, SAPS II may be influenced by various sources of bias, such as differences in patient case-mix, variations in data quality or availability, or differences in clinical practice patterns across different hospitals or regions. These factors could limit the generalizability and accuracy of the score in some settings. Fourthly, SAPS II was developed and validated using data from the 1990s, and the characteristics and treatment options of ICU patients may have changed since then. This could limit the temporal applicability of the score and its ability to accurately predict outcomes in modern ICU patients. Finally, while SAPS II can provide useful information about a patient's mortality risk, it should not be used in isolation or as the sole criterion for clinical decision-making. Other factors, such as clinical judgment, patient preferences, and available treatment options, should also be considered when making treatment decisions for critically ill patients.

Overall, while SAPS II is a useful and widely used tool for predicting mortality risk in ICU patients, there is still room for improvement in its accuracy and usefulness. At the same time, it is important to recognize its limitations and to use it in conjunction with other clinical information and judgment.

## Conclusions

To summarize, the findings of the present study provide valuable insights into the performance of different severity assessment scores (APACHE II, SAPS II, SOFA, and REMS) in predicting mortality in ICU patients. Both SAPS II and APACHE II were observed to have good calibration and discriminatory power; however, SAPS II had the best prediction power suggesting that it may be a useful tool for clinicians and researchers in assessing the severity of illness and mortality risk in critically ill patients. Nevertheless, further studies are needed to confirm these findings and to explore the limitations and potential biases of SAPS II and other severity assessment scores.
